# Real-World Dual Antiplatelet Therapy Following Polymer-Free Sirolimus-Eluting Stent Implantations to Treat Coronary Artery Disease

**DOI:** 10.1007/s10557-020-06963-5

**Published:** 2020-03-24

**Authors:** Florian Krackhardt, Matthias Waliszewski, Viktor Kočka, Petr Toušek, Bronislav Janek, Martin Hudec, Fernando Lozano, Koldobika Garcia-San Roman, Bruno Garcia del Blanco, Josepa Mauri, Tay Mok Heang, Tae Hoon Ahn, Myung Ho Jeong, Denny Herberger, Vjekoslav Tomulic, Gilles Levy, Laurent Sebagh, Jérôme Rischner, Michel Pansieri

**Affiliations:** 1 Department of Internal Medicine and Cardiology, Charité – Universitätsmedizin Berlin, Campus Virchow, Augustenburger Platz 1, D-13353 Berlin, Germany; 2 Medical Scientific Affairs, B.Braun Melsungen AG, Berlin, Germany; 3 University Hospital Královské Vinohrady, Prague, Czech Republic; 4 IKEM, Prague, Czech Republic; 5 SÚSCCH, a.s. Banská Bystrica, Slovak Republic; 6 Hospital General Universitario de Ciudad Real, Ciudad Real, Spain; 7 Hospital Universitario de Cruces, Bilbao, Spain; 8 Hospital Universitari Vall d’Hebron Barcelona, Barcelona, Spain; 9 Hospital Universitari Germans Trias i Pujol, Badalona, Spain; 10 Pantai Ayer Keroh Hospital, Malacca, MLK Malaysia; 11 Gachon University Gil Medical Center, Incheon, South Korea; 12 Chonnam National University, Gwangju, South Korea; 13 Clinical Hospital Center Rijeka, Rijeka, Croatia; 14 Clinique du Millénaire, Montpellier, France; 15 Clinique Turin Paris, Paris, France; 16 Hôpital Albert Schweitzer Colmar, Colmar, France; 17 Centre Hospitalier d’Avignon, Avignon, France

**Keywords:** Dual antiplatelet therapy, Clopidogrel, Ticagrelor, Polymer-free, Sirolimus-eluting stent

## Abstract

**Objectives:**

The objective of this post hoc analysis was to analyze real-world dual antiplatelet therapy (DAPT) regimens following polymer-free sirolimus-eluting stent (PF-SES) implantations in an unselected patient population.

**Methods:**

Patient-level data from two all-comers observational studies (ClinicalTrials.gov Identifiers: NCT02629575 and NCT02905214) were pooled and analyzed in terms of their primary endpoint. During the data verification process, we observed substantial deviations from DAPT guideline recommendations. To illuminate this gap between clinical practice and guideline recommendations, we conducted a post hoc analysis of DAPT regimens and clinical event rates for which we defined the net adverse event rate (NACE) consisting of target lesion revascularization (TLR, primary endpoint of all-comers observational studies) all-cause death, myocardial infarction (MI), stent thrombosis (ST), and bleeding events. A logistic regression was utilized to determine predictors why ticagrelor was used in stable coronary artery disease (CAD) patients instead of the guideline-recommended clopidogrel.

**Results:**

For stable CAD, the composite endpoint of clinical, bleeding, and stent thrombosis, i.e., NACE, between the clopidogrel and ticagrelor treatment groups was not different (5.4% vs. 5.1%, *p* = 0.745). Likewise, in the acute coronary syndrome (ACS) cohort, the NACE rates were not different between both DAPT strategies (9.2% vs. 9.3%, *p* = 0.927). There were also no differences in the accumulated rates for TLR, myocardial infarction ([MI], mortality, bleeding events, and stent thrombosis in elective and ACS patients. The main predictors for ticagrelor use in stable CAD patients were age < 65 years, smaller vessels, treatment of ostial and calcified lesions, and in-stent restenosis.

**Conclusion:**

Within the framework of a post hoc analysis based on a real-world, large cohort study, there were no differences in the combined endpoint of major adverse cardiac events (MACE), bleeding and thrombotic events for clopidogrel and ticagrelor in stable CAD or ACS patients. Despite the recommendation for clopidogrel by the European Society of Cardiology (ESC), real-world ticagrelor use was observed in subgroups of stable CAD patients that ought to be explored in future trials.

## Introduction

**Fig. 1:**
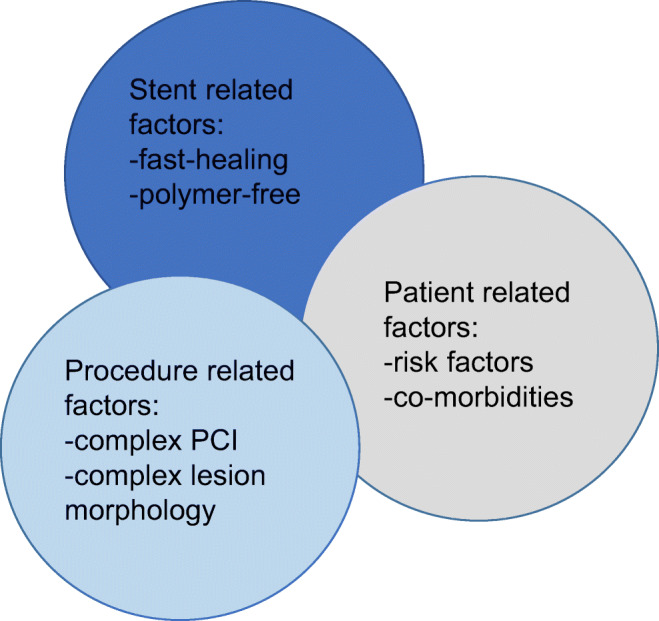
Potential factors for clinical events and stent thrombosis following DES implantations, modified after to Byrne et al. [2]

The current European Society of Cardiology (ESC) guidelines [1] provide recommendations for dual antiplatelet therapy (DAPT) following percutaneous coronary interventions (PCIs). However, this guidance to balance the risks for ischemic events and bleeding episodes provide some latitude. However, in stable coronary artery disease (CAD), there is only one recommended regimen consisting of clopidogrel and aspirin. Since the “ischemic” risks for major cardiac events (MACE) in particular for stent thrombosis (ST) following drug-eluting stent (DES) implantations are multifactorial [2], it is important to control as many of these factors as possible (Fig. 1). Therefore, it seems advantageous to study these risks in a large patient population which received one particular DES to eliminate stent-related factors. In this case, the effect of DAPT on the outcomes can be better elucidated. Nevertheless, previous studies focused either on different devices (bare metal stents [BMS] vs. DES) and/or different DAPT modalities (duration).

Urban et al. [3] focused on stent-related factors and compared a drug-coated stent to a BMS in patients with high bleeding risk (HBR) while both groups had the same DAPT regimen. They reported that the drug-coated stent with only 1 month of DAPT was superior to its BMS analogue in terms of safety (cardiac death, myocardial infarction, and ST) and efficacy relative to target lesion revascularization (TLR). While this milestone randomized controlled trial (RCT) investigated the risk/benefit ratio of bleeding vs. ischemic events with a drug-coated stent in high-risk patients, other researchers felt also inspired to study shorter DAPT durations in other clinical scenarios.

In the SENIOR trial [4], the study of patient related factors was the objective. Elderly patients with an increased bleeding risk were randomized in two groups to receive either a DES or BMS while the type of DAPT was not specified, i.e., all P2Y12 receptor inhibitors were permissible. A very comprehensive overview of the type of DAPT was given in the ESC guidelines updated in 2017 [1]. In these guidelines, neither ticagrelor nor prasugrel were recommended for patients with stable coronary artery disease (CAD).

The meta-analysis conducted by Palmerini and coworkers [5], once again a mélange of different DAPT agents and various DES technologies, revealed that in stable CAD patients, there were no significant differences in terms of myocardial infarction (MI) and ST, cardiac death, or any bleeding rates between a short and regular DAPT duration (3 vs. 6 months).

Given the favorable clinical outcomes of a polymer-free thin strut sirolimus-eluting stent (PF-SES) in unselected non-HBR patients [6, 7] with postulated rapid strut coverage [8], we decided to pool data from two observational studies having an identical protocol to analyze the data of a large cohort. During the data analysis, we realized that ticagrelor was used even in elective patients. This in turn triggered subsequent analyses why this more aggressive P2Y12 receptor blocker was used in this particular patient group. Due to the fact that sufficiently large patient cohorts were available and only one particular DES was used, we could study the effect of either aspirin + clopidogrel or aspirin + ticagrelor following in patients with stable CAD and acute coronary syndrome (ACS).

Our primary objective was to study these two DAPT regimens (aspirin + clopidogrel or aspirin + ticagrelor) after PF-SES implantation with a defined post hoc composite endpoint, i.e., net adverse coronary event (NACE) defined as the cumulative event rates of TLR, MI, all-cause mortality, ST, and bleeding rates in “real-world” patients.

## Methods

### End Points and Definitions

The international ISAR 2000 all-comers registry (ClinicalTrials.gov Identifier NCT02629575) [6, 7] and the ISAR 2000 all-comers extended registry (ClinicalTrials.gov Identifier NCT02905214) prospectively enrolled patients in Europe and Asia. Prior to patient recruitment, all relevant ethics committees approved the study protocol. To accommodate for national differences of follow-up windows, mainly due to reimbursement issues, a timeframe of 9–12 months was permissible. In the original two studies, target lesion revascularization rate (TLR, coronary artery bypass grafting and Re-PCI) at follow-up was the primary end point, whereas the rate of MACE and the corresponding rates of myocardial infarction (MI) were part of the secondary endpoints. Cardiac death was only defined in-hospital whereas the all-cause death rate was used to define MACE at 9–12 months (MI, TLR, in-hospital cardiac death, all deaths post discharge).

During the data verification process, we observed substantial deviations from the DAPT guideline recommendations. To illuminate this gap between clinical practice and guideline recommendation, we conducted a post hoc analysis of all patients receiving either clopidogrel or ticagrelor. To account for ischemic and bleeding events, we defined the net adverse event rate (NACE) which was based on the KAMIR-NIH study conducted by Sim and coworkers [9]. NACE is a composite endpoint consisting of target lesion revascularization (TLR, primary endpoint of all-comers observational studies) all-cause death, myocardial infarction (MI), stent thrombosis (ST), and bleeding events. The definition of acute/subacute stent thromboses (ST) was based on the ARC criteria [10]. Bleeding events were defined according to the BARC classification [11] whereas major bleeding episodes were collectively defined as BARC 3a-5.

A glomerular filtration rate (GFR) < 90 mL/min/1.73m^2^ defined renal insufficiency while the cut-off GFR rate for mandatory dialysis was < 15 mL/min/1.73 m^2^. Severe tortuous vessels were defined by the angulation criterion of > 45°.

### Centers

Patients were prospectively enrolled in 39 Asian (South Korea, Malaysia) and 43 European (Croatia, Czech Republic, France, Germany, Slovakia, Spain) cardiac centers.

### Materials

All patients received PF-SES of identical polymer-free coating consisting of probucol and sirolimus (Coroflex© ISAR or Coroflex© ISAR NEO, B.Braun Melsungen AG, Germany). All PF-SES were implanted in accordance with each institution’s guidelines and preferences. The PF-SES was described in detail by Krackhardt et al. [6].

### Inclusion and Exclusion Criteria

Adult patients with stable angina and objective proof of ischemia or patients with acute coronary syndrome (ACS) had to meet the requirements for PCI at the time the study was being conducted [12]. Stenting was allowed in de novo or restenotic lesions of single or multiple vessels with reference diameters from 2.0 to 4.0 mm.

### Procedural Approach

Radial or femoral vascular access was permitted with a recommended introducer sheath of at least five French in diameter. Pre-dilatation with a balloon catheter of the operators’ preference or the direct stenting approach could be chosen. Intravenous heparin (70 IU/kg) was given in all patients and supplemented as needed. According to the institutional preferences of the cardiac centers, platelet aggregation inhibitor loading was recommended but not mandatory.

### Post-Procedural Medication

Due to the all-comers nature of this assessment which encompassed centers from Europe and Asia, the choice and duration of the P2Y12 receptor inhibitor was defined by the ESC guideline [12], i.e., 6 months of clopidogrel for patients with stable CAD, and 12 months for ACS patients. As previously reported by Krackhardt et al. [6], various antiplatelet inhibition agents (≥ 6 months) such as clopidogrel 75 mg/day, prasugrel 10 mg/day, or ticagrelor 2 × 90 mg/day were allowed in conjunction with acetylsalicylic acid 100–325 mg/day life long as recommended by the treating physician.

### Data Collection

An electronic data capture system [13, 14] was used which has built-in plausibility checks during each stage of the data entry. Each participating country had a national principal investigator who verified the accuracy of the dataset on a national level whenever routinely performed web-based plausibility checks indicated discrepancies.

### Statistical Analysis

Continuous variables are expressed in means and standard deviations and compared with the unpaired *t* test or the Mann-Whitney *U* test in case the Shapiro-Wilk test revealed a strong deviation from a normal distribution. Dichotomous and categorical variables are described in counts and percentages and evaluated with the two-sided Fisher’s exact test or the chi^2^ statistic whenever applicable.

Moreover, a logistic regression with various covariates (patient, lesion and procedural parameters) was conducted with “ticagrelor use” as the dependent variables. This post hoc analysis was done to study “predictors for ticagrelor use” in elective patients. The significance level α was 0.05 for all tests. SPSS version 24.0 (IBM, Munich, Germany) was used for all statistical analyses.

### Ethics Approval

Prior to patient recruitment, all ethics votes were obtained from relevant national and/or local ethics committees. In France, these non-interventional studies were approved by the *Comité Consultative sur le Traitement de l’Information en matière de Recherche dans le domaine de la Santé* (CCTIRS dossier no. 14.613) and the *Commission Nationale de l’informatique et des Libertés* (CNIL, demande d’autorisation n°915,019). This study was conducted within the framework of the Declaration of Helsinki in its most current form.

## Results

### Total Study Population

**Fig. 2:**
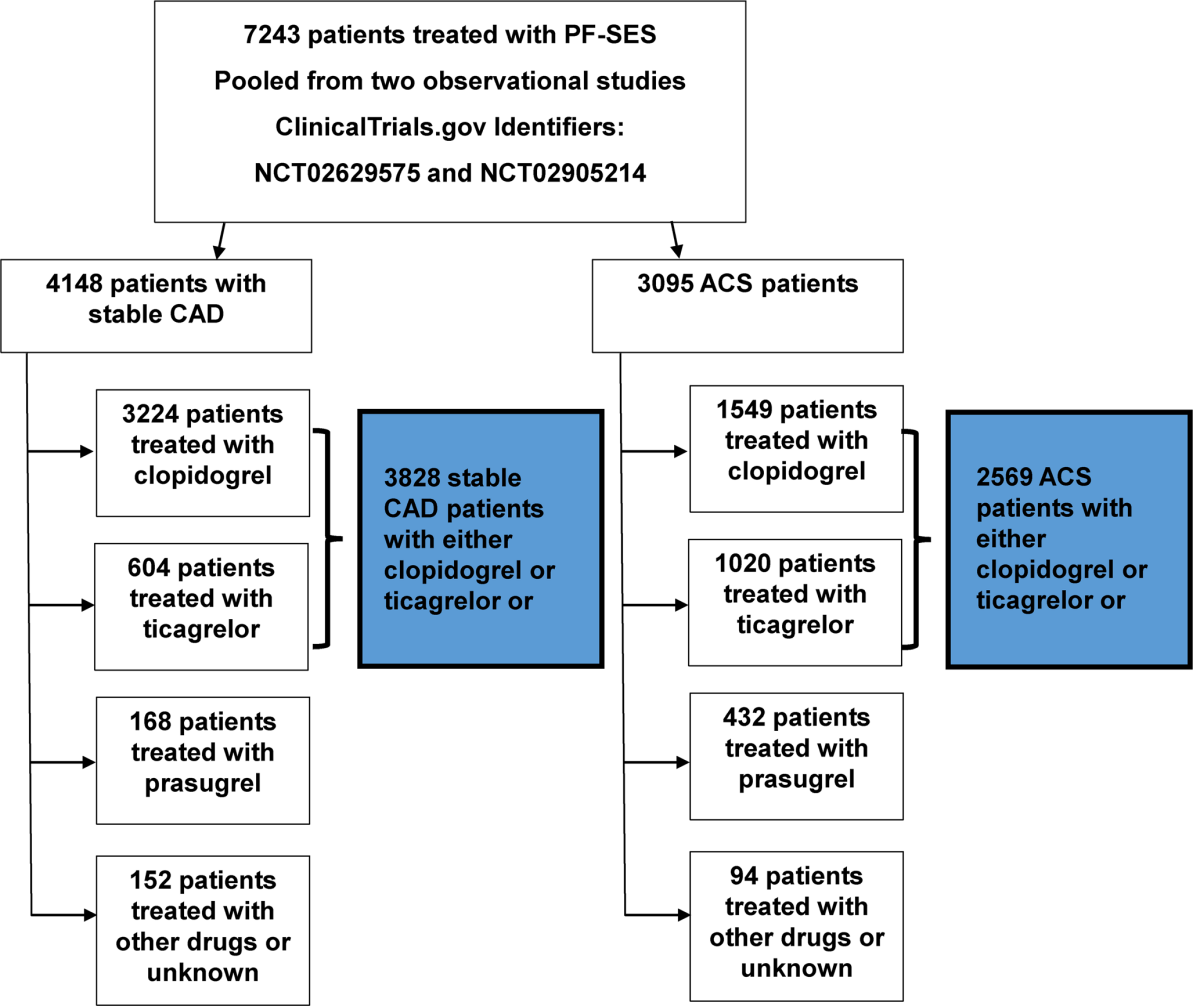
Post hoc analysis selection of patients with stable CAD or ACS, who were treated with clopidogrel or ticagrelor

Between November 2014 and December 2017, 7243 patients were enrolled and treated with PF-SES. Patient demographics, lesion morphologies, and procedural details are detailed in Table 1 for those patients who received either clopidogrel or ticagrelor. Of these, 3828 patients had stable CAD and were treated either with clopidogrel (3224, 84.2%) or ticagrelor (604, 15.8%). Likewise in the ACS subgroup, a total of 2569 patients (clopidogrel 1549, 60.3% vs. ticagrelor: 1020, 29.7%) were available for analysis. Patients who were treated with either clopidogrel (*n* = 3224) or ticagrelor (*n* = 604) were used for further analyses (Fig. 2).

**Table 1:** Patient demographic data, lesion morphologies, and procedural details in patients treated with clopidogrel or ticagrelor after treatment with polymer-free sirolimus-eluting stents

Variable	Stable CAD			ACS		
	Clopidogrel	Ticagrelor	*p* value	Clopidogrel	Ticagrelor	*p* value
Number of patients	3224	604	–	1549	1020	
Number of lesions	3581	708	–	1711	1150	
Number of DES used	3954	785	–	1825	1229	
Age (years)	68.1 ± 10.5	64.5 ± 10.3	<0.001	68.1 ± 12.7	63.9 ± 11.3	< 0.001
Male gender	2346 (72.8%)	465 (77.0%)	0.031	1104 (71.3%)	795 (77.9%)	< 0.001
Diabetes	1294 (40.1%)	222 (36.8%)	0.119	590 (38.1%)	297 (29.1%)	< 0.001
Hypertension	2422 (75.1%)	395 (65.4%)	<0.001	1083 (69.9%)	606 (59.4%)	< 0.001
Renal insufficiency	241 (7.5%)	26 (4.3%)	0.005	117 (7.6%)	47 (4.6%)	0.003
Dialysis dependence	66 (2.0%)	6 (1.0%)	0.080	12 (0.8%)	6 (0.6%)	0.579
Region						
Europe	2383 (73.9%)	476 (78.8%)	0.011	1186 (76.6%)	873 (85.6%)	< 0.001
Asia	841 (26.1%)	128 (21.2%)		363 (23.4%)	147 (14.4%)	
Target vessel						
LAD	1490 (41.6%)	317 (44.8%)	0.054	751 (43.9%)	500 (43.5%)	0.546
CX	962 (26.9%)	198 (28.0%)		431 (25.2%)	269 (23.4%)	
RCA	1093 (30.5%)	191 (27.0%)		200 (31.4%)	173 (26.0%)	
graft	36 (1.0%)	2 (0.3%)		15 (0.9%)	10 (0.9%)	
Thrombotic occlusion	224 (6.3%)	43 (6.1%)	0.855	326 (19.1%)	261 (22.7%)	0.018
Chronic total occlusion	139 (3.9%)	30 (4.2%)	0.657	38 (2.2%)	20 (1.7%)	0.370
Diffuse vessel disease	1465 (40.9%)	280 (39.5%)	0.500	693 (40.5%)	427 (37.1%)	0.070
Calcification	1040 (29.0%)	215 (30.4%)	0.479	572 (33.4%)	272 (23.7%)	< 0.001
Ostial lesion	283 (7.9%)	79 (11.2%)	0.004	146 (8.5%)	79 (6.9%)	0.105
Bifurcations	501 (14.0%)	87 (12.3%)	0.229	263 (15.4%)	162 (14.1%)	0.344
In-stent restenosis	117 (3.3%)	34 (4.8%)	0.043	45 (2.6%)	28 (2.4%)	0.745
Severe tortuosity	371 (10.4%)	53 (7.5%)	0.019	206 (12.0%)	88 (7.7%)	< 0.001
Saphenous vein graft	36 (1.0%)	2 (0.3%)	0.061	15 (0.9%)	10 (0.9%)	0.984
AHA/ACC type B2/C lesion	1833 (51.3%)	346 (48.9%)	0.260	969 (56.6%)	636 (55.3%)	0.482
Reference diameter (mm)	2.87 ± 0.48	2.82 ± 0.48	0.042	2.86 ± 0.52	2.90 ± 0.54	0.034
Lesion length	18.4 ± 9.5	18.9 ± 10.6	0.231	18.9 ± 8.8	18.3 ± 8.3	0.083
Degree of stenosis (%)	84.1 ± 11.0	83.1 ± 12.2	0.042	90.0 ± 11.2	89.3 ± 11.1	0.484
Predilation	2413 (67.4%)	439 (62.0%)	0.006	1249 (73.0%)	766 (66.6%)	< 0.001
DESs used	3954	785	–	1825	1229	
Multi-vessel PCI						
1-vessel	3022 (93.6%)	557 (92.2%)	0.194	1462 (94.4%)	939 (92.1%)	0.060
2-vessel	193 (6.0%)	42 (7.0%)		79 (5.1%)	75 (7.4%)	
3-vessel	12 (0.4%)	5 (0.8%)		8 (0.5%)	6 (0.6%)	
DES per patient	1.25 ± 0.64	1.32 ± 0.69	0.014	1.21 ± 0.58	1.28 ± 0.65	0.001
DES diameter (mm)	2.86 ± 0.48	2.81 ± 0.47	0.006	2.84 ± 0.53	2.88 ± 0.53	0.021
DES length (mm)	20.8 ± 8.7	20.8 ± 8.5	0.862	21.1 ± 7.6	20.7 ± 7.7	0.196
DES inflation pressure (atm)	14.1 ± 2.9	14.1 ± 2.8	0.746	14.7 ± 2.9	14.5 ± 2.7	0.011
Overall technical success per stent	3899 (98.6%)	777 (99.0%)	0.406	1789 (98.0%)	1214 (98.8%)	0.112
Patients with follow-up	2909 (90.2%)	550 (91.1%)	0.526	1375 (88.8%)	873 (85.6%)	0.017
DAPT duration in months	9.8 ± 2.9	10.4 ± 2.7	<0.001	10.9 ± 2.4	11.3 ± 2.0	< 0.001
DAPT ≤ 3 months	76 (2.4%)	11 (1.8%)	0.417	37 (2.4%)	12 (1.2%)	0.028
Triple therapy	65 (2.0%)	1 (0.1%)	0.001	28 (1.8%)	5 (0.5%)	0.004

Overall, elective patients receiving ticagrelor in stable CAD were more frequently treated in ostial lesions (11.1% vs. 6.8%, *p* = 0.006) and had longer DAPT (10.5 ± 2.6 months vs. 9.9 ± 2.8 months, *p* < 0.001) and were less frequent on triple therapy (0.1% vs. 1.7%, *p* = 0.006). Besides the above-mentioned differences, procedural and lesion related characteristics in the stable CAD groups were reasonably similar (Table 2). In terms of clinical event rates, there were no significant differences between patients treated with either clopidogrel or ticagrelor in terms of NACE (5.4% vs. 5.1%, *p* = 0.745), MACE (2.8% vs. 3.3%), thrombotic events (0.5% vs. 0.9%, *p* = 0.213), or bleeding episodes (2.8% vs. 2.2%, *p* = 0.399).

**Table 2:** Clinical outcomes in patients treated with clopidogrel or ticagrelor and polymer-free sirolimus-eluting stents

Variable	Stable CAD			ACS		
	Clopidogrel	Ticagrelor	*p* value	Clopidogrel	Ticagrelor	*p* value
Number of patients	3224	604	–	1549	1020	–
Patients with clinical long term follow-up or early event	2909 (90.2%)	550 (91.1%)	0.526	1375 (88.8%)	873 (85.6%)	0.017
Follow-up time (months)	9.3 ± 2.0	9.2 ± 2.2	0.058	9.3 ± 2.5	9.3 ± 2.4	0.961
Time to discharge (days)	3.9 ± 20.8	2.6 ± 10.6	0.034	3.3 ± 3.7	3.2 ± 3.1	0.473
Accumulated NACE	158 (5.4%)	28 (5.1%)	0.745	126 (9.2%)	81 (9.3%)	0.927
Accumulated MACE	80 (2.8%)	18 (3.3%)	0.498	83 (6.0%)	47 (5.4%)	0.518
Accumulated TLR	50 (1.7%)	9 (1.6%)	0.891	37 (2.7%)	19 (2.2%)	0.446
Re-PCI	43 (1.5%)	9 (1.6%)	0.780	36 (2.6%)	16 (1.8%)	0.227
CABG	9 (0.3%)	0 (0.0%)	0.191	5 (0.4%)	8 (0.9%)	0.092
Accumulated MI	16 (0.6%)	7 (1.3%)	0.056	25 (1.8%)	22 (2.5%)	0.257
Accumulated death all causes	22 (0.8%)	5 (0.9%)	0.709	39 (2.8%)	14 (1.6%)	0.060
Accumulated definite/ probable stent thrombosis	14 (0.5%)	5 (0.9%)	0.213	8 (0.6%)	7 (0.8%)	0.532
Acute stent thrombosis, ≤ 24	5 (0.2%)	3 (0.5%)	0.241	4 (0.1%)	3 (0.3%)	0.351
Subacute stent thrombosis,1–30 days	0 (0.0%)	0 (0.0%)		2 (0.1%)	0 (0.0%)	
Late stent thrombosis, ≥ 30 days	9 (0.3%)	2 (0.4%)		2 (0.1%)	4 (0.5%)	
Bleeding complications	82 (2.8%)	12 (2.2%)	0.399	46 (3.3%)	37 (4.2%)	0.274
Minor	69 (2.4%)	9 (1.6%)	0.287	38 (2.8%)	32 (3.7%)	0.230
Major	13 (0.4%)	3 (0.5%)	0.755	8 (0.6%)	5 (0.6%)	0.978

In the ACS cohort, the NACE rates were significantly higher than the corresponding rates in elective patients (clopidogrel: p_stable CAD vs. ACS_<0.001, ticagrelor: p_stable CAD vs. ACS_=0.031). Neither MACE, TLR, bleeding, or thrombotic events were significantly different in ACS patients treated with either clopidogrel or ticagrelor. However, the accumulated mortality rate trended higher in the clopidogrel group as compared to the ticagrelor group (2.8% vs. 1.6%, *p* = 0.060).

### Predictors for Ticagrelor Use in Stable CAD

**Fig. 3:**
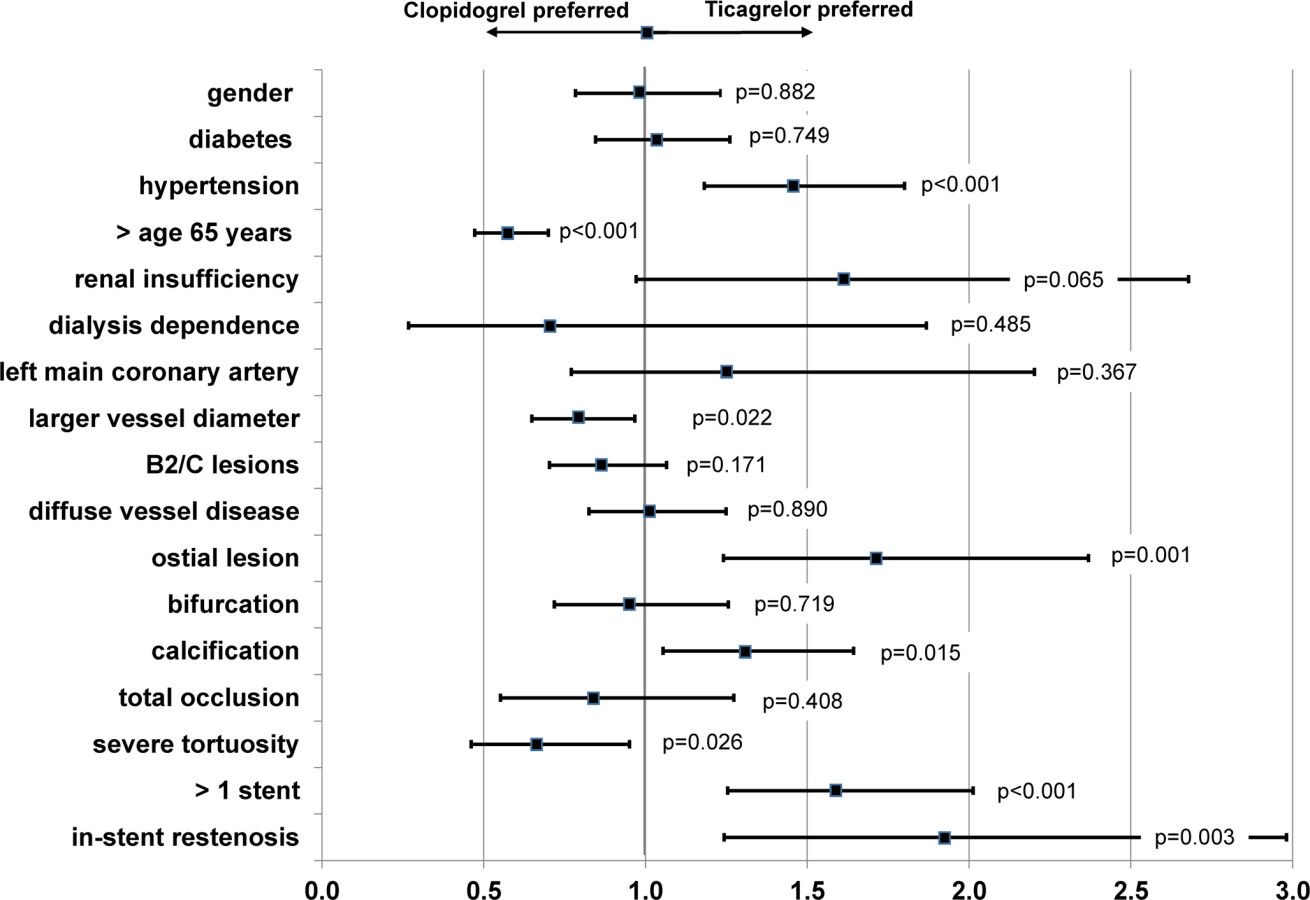
Forest plot and odds ratios for ticagrelor use for various covariates

The forest plot of the logistic regression analysis is shown in Fig. 3. Predictors for ticagrelor use were patients < 65 years of age (*p* < 0.001), hypertension (*p* < 0.001), smaller reference vessels (*p* = 0.022), stenting in ostial lesions (*p* = 0.001), presence of calcification (*p* = 0.015), the use of more than one stent (*p* < 0.001), and in-stent restenosis (*p* = 0.003).

### DAPT Length and Follow-Up Duration

A post hoc analysis of DAPT durations was also conducted with the pooled clopidogrel/ticagrelor data (Table 3). Based on an established cut-off value of ≤ 3 months [1], there were no differences between the short DAPT (≤ 3 months) and the long DAPT regimen (> 3 months) in terms of NACE, MACE, TLR, all-cause death, and bleeding complications up to the follow-up. The accumulated MI rates between these two groups, however, were borderline significant (0.6% vs. 2.5%, *p* = 0.043). The time to discharge was shorter in patients that had ≤ 3 months of DAPT as compared to those with > 3 months (3.4 ± 10.6 vs. 3.9 ± 20.6, *p* = 0.030). The shorter DAPT duration was strongly associated with triple therapy (11.5% vs. 1.5%, *p* < 0.001).

**Table 3:** Clinical outcomes in patients with stable CAD on short and long DAPT with either clopidogrel or ticagrelor

Variable	> 3 months	≤ 3 months	*p* value
Number of patients	3741	87	–
Patients on triple therapy	56 (1.5%)	10 (11.5%)	< 0.001
Patients with clinical long term follow-up or early event	3378 (90.3%)	81 (93.1%)	0.381
Follow-up time (months)	9.3 ± 2.0	8.8 ± 2.0	0.825
Time to discharge (days)	3.9 ± 20.6	3.4 ± 10.6	0.030
Accumulated NACE	181 (5.4%)	5 (6.2%)	0.748
Accumulated MACE	96 (2.8%)	2 (2.5%)	0.842
Accumulated TLR	58 (1.7%)	1 (1.2%)	0.740
Re-PCI	51 (1.5%)	1 (1.2%)	0.841
CABG	8 (0.2%)	1 (1.2%)	0.082
Accumulated MI	21 (0.6%)	2 (2.5%)	0.043
Accumulated death all causes	27 (0.8%)	0 (0.0%)	0.419
Accumulated definite/probable stent thrombosis	18 (0.5%)	1 (1.2%)	0.398
Acute stent thrombosis, ≤ 24	8 (0.2%)	0 (0.0%)	0.303
Subacute stent thrombosis,1–30 days	0 (0.0%)	0 (0.0%)	
Late stent thrombosis, ≥ 30 days	10 (0.3%)	1 (1.2%)	
Bleeding complications	92 (2.7%)	2 (2.5%)	0.889
Minor	77 (2.3%)	1 (1.2%)	0.531
Major	15 (0.4%)	1 (1.2%)	0.300

A follow-up window of 9–12 months was requested by several European countries (e.g., Belgium) due to local reimbursement requirements. The “a priori” defined subgroup with follow-up data ≥ 12 months was analyzed to better respond to the documentation needs in these countries. There was a total of 264 patients with a follow-up duration (≥ 12 months), including premature events, of 13.3 ± 2.8 months. The corresponding NACE rate in the longer follow-up group was 6.6% (33/501) with no differences between the DAPT groups (clopidogrel 5.9% vs. ticagrelor 8.4%, *p* = 0.303). Further analyses revealed that the accumulated MACE rate in the longer follow-up group was 4.0% (20/501) without differences between patients receiving clopidogrel and ticagrelor (3.1% vs. 6.3%, *p* = 0.096). However, the MI rates were significantly different between both subgroups (0.8% vs. 4.9%, *p* = 0.003) with an overall MI rate of 2.0% (10/501). In the longer follow-up cohort, the accumulated mortality rate was 0.6% (3/501) without differences between DAPT groups (0.8% vs. 0.0%, *p* = 0.272). Likewise there was no difference in the accumulated rates for definite/probably stent thrombosis with an overall rate of 0.9% (4/501) and without differences in patients receiving clopidogrel or ticagrelor (0.6% vs. 1.5%, *p* = 0.335).

## Discussion

There is a plethora of potential factors and confounders (Fig. 1), which may determine the risk for ischemic events and bleeding episodes in patients undergoing stenting procedures. In the updated ESC guidelines [1], ticagrelor and prasugrel are not recommended for patients with stable CAD. Nevertheless, these new P2Y12 receptor inhibitors are being prescribed for stable CAD patients in clinical practice.

### Use of Ticagrelor in Stable CAD Patients

The use of new P2Y12 receptor inhibitors (ticagrelor, prasugrel) is only recommended in ACS patients with a class I a recommendation. However, despite the data granularity of our all-comers observational study of close to 3800 stable CAD patients, 15.8% of these patients treated were treated with ticagrelor in real-world clinical practice. But why were these patients not treated with clopidogrel? One could speculate that the use of a polymer-free DES and the concomitant prescription of a more effective antiplatelet agent such as ticagrelor followed a “belt and suspenders” strategy in patients with low/moderate bleeding risk. Our regression analysis revealed that ticagrelor is frequently used in stable CAD patients of younger age and/or lesions with smaller vessel diameters and ostial and calcified lesions. In other words, these attributes may have been considered more challenging in terms of their cardiovascular risk factors or culprit lesion morphology. The higher rate of ostial lesions in the ticagrelor DAPT group (11.2% vs. 7.9%, *p* = 0.004) may serve as an explanation for this theory. The treatment of smaller vessels (*p* = 0.022) and in-stent restenosis (*p* = 0.003) support this theory whereas patients with severe vessel tortuosity received more often clopidogrel (*p* = 0.026).

Moreover, patients in the ticagrelor group were less frequent on triple therapy as compared to those who had clopidogrel as part of their standard DAPT (0.1% vs. 2.0%, *p* = 0.001). Patients on oral anticoagulation were preferably treated with clopidogrel probably due to its safety profile in this subgroup.

Finally, despite our clinical outcomes in stable CAD patients who were treated with ticagrelor, it is not our intention to promote an off-label use but to merely stimulate critical discussion beneficial for a potential trial design if this strategy were deemed worthwhile.

### Safety and Bleeding Episodes

In our stable CAD cohort, there were no differences in minor and major bleeding episodes (2.8% vs. 2.2%, *p* = 0.399). However, in the ticagrelor DAPT group, the acceptably low bleeding rate may be explained by the significantly lower frequency of patients on triple therapy.

In the COMPASS trial [15], a different, more aggressive pharmacotherapeutic strategy was investigated in patients with coronary and/or peripheral artery disease with the same objective to reduce cardiovascular event rates. Three groups of patients who received different treatment modalities consisting of aspirin and rivaroxaban were studied. The authors concluded that the rates for the composite primary endpoint were significantly lower in the more aggressive low-dose rivaroxaban plus aspirin treatment groups. This anticoagulant/antithrombotic approach is in line with our observed strategy to use the more effective ticagrelor + aspirin DAPT strategy for those patients and procedures with a low risk of bleeding events.

### DES or Co-Medication?

It seems that differences between modern generation DES technologies are becoming less important. Acceptably low ST rates and clinical event rates are reported with various DES platforms [16–18]. Therefore, the type of DAPT and its duration chosen on the basis of patient and procedure-related factors are likely to determine the frequency of cardiovascular events. Further enhancements in optimizing the risk/benefit ratio of bleeding vs. ischemic events are determined by an objective algorithm to choose the right patient and lesion with short and/or more aggressive DAPT. The call for DAPT customization is, albeit its renewed interest first initiated by the LEADERS FREE trial [3], not new and was already proposed by Pfisterer et al. [19]. We suspect that our results indicate that the observed ticagrelor use in stable CAD patients suggests a well-balanced benefit/risk ratio for younger patients and those with ostial lesions, calcified lesions within the limitations of our study.

### Limitations

Within the nature of an observational study of this size, there is data granularity which can be viewed in terms of event underreporting, real-world DAPT modifications during follow-up, and PCI of other vessels following PF-SES implantations just to name a few. To assure that patients not available for clinical follow-up did not have a higher risk profile in terms of lesion morphology and cardiovascular risk factors, we conducted a chi^2^ analysis. This did not reveal that patients lost to follow-up had a higher risk profile. We did not attempt to filter those patients who were converted from ticagrelor to clopidogrel for symptoms of dyspnea. This may have introduced some unknown bias. A classification of the vascular access route (femoral vs. radial) was not within the scope of this clinical assessment and could have had an effect on the severity of post-procedural access site bleeding. This could have left one predictor for access site bleeding events uncovered. Our findings are hypothesis generating and not meant to suggest ticagrelor use in elective patients. Finally, we only had an observational period of 9–12 months which is too short to account for late ischemic events.

## Conclusions

There were no differences in either stable CAD or ACS patients treated with either clopidogrel or ticagrelor in terms of NACE or any other clinical event rates. Ticagrelor is frequently used in stable CAD patients of younger age and those having lesions with smaller vessel diameters and/or other lesion morphologies (ostial lesions, calcification, in-stent-restenosis).

The selection process for patients and lesions suitable for ticagrelor DAPT in stable CAD should be further studied in larger dedicated trials with a special focus on our predictors for ticagrelor use.

## Data Availability

The datasets generated during and/or analyzed during the current study are not publicly available due proprietary reasons and data protection reasons as stated in the protocol but are available from the corresponding author on reasonable request.
