# Correction to: Real-World Dual Antiplatelet Therapy Following Polymer-Free Sirolimus-Eluting Stent Implantations to Treat Coronary Artery Disease

**DOI:** 10.1007/s10557-021-07290-z

**Published:** 2022-07-22

**Authors:** Florian Krackhardt, Matthias Waliszewski, Viktor Kočka, Petr Toušek, Bronislav Janek, Martin Hudec, Fernando Lozano, Koldobika Garcia-San Roman, Bruno Garcia del Blanco, Josepa Mauri, Tay Mok Heang, Tae Hoon Ahn, Myung Ho Jeong, Denny Herberger, Vjekoslav Tomulic, Gilles Levy, Laurent Sebagh, Jérôme Rischner, Michel Pansieri

**Affiliations:** 1 Department of Internal Medicine and Cardiology, Charité - Universitätsmedizin Berlin, Campus Virchow, Augustenburger Platz 1, D-13353 Berlin, Germany; 2 Medical Scientific Affairs, B.Braun Melsungen AG, Berlin, Germany; 3 University Hospital Královské Vinohrady, Prague, Czech Republic; 4 IKEM, Prague, Czech Republic; 5 SÚSCCH, a.s. Banská, Bystrica, Slovak Republic; 6 Hospital General Universitario de Ciudad Real, Ciudad Real, Spain; 7 Hospital Universitario de Cruces, Bilbao, Spain; 8 Hospital Universitari Vall d’Hebron Barcelona, Barcelona, Spain; 9 Hospital Universitari Germans Trias i Pujol, Badalona, Spain; 10 Pantai Ayer Keroh Hospital, Malacca, MLK Malaysia; 11 Gachon University Gil Medical Center, Incheon, South Korea; 12 Chonnam National University, Gwangju, South Korea; 13 Clinical Hospital Center Rijeka, Rijeka, Croatia; 14 Clinique du Millénaire, Montpellier, France; 15 Clinique Turin Paris, Paris, France; 16 Hôpital Albert Schweitzer Colmar, Colmar, France; 17 Centre Hospitalier d’Avignon, Avignon, France

**Correction to: Cardiovascular Drugs and Therapy (2021)**

10.1007/s10557-020-06963-5

The article “Real-World Dual Antiplatelet Therapy Following Polymer-Free Sirolimus-Eluting Stent Implantations to Treat Coronary Artery Disease” written by Florian Krackhardt, Matthias Waliszewski, Viktor Kočka, Petr Toušek, Bronislav Janek, Martin Hudec, Fernando Lozano, Koldobika Garcia-San Roman, Bruno Garcia del Blanco, Josepa Mauri, Tay Mok Heang, Tae Hoon Ahn, Myung Ho Jeong, Denny Herberger, Vjekoslav Tomulic, Gilles Levy, Laurent Sebagh, Jérôme Rischner, and Michel Pansieri was originally published Online First without Open Access. After publication, the author decided to opt for Open Choice and to make the article an Open Access publication. Therefore, the copyright of the article has been changed to © The Author(s) 2021 and the article is forthwith distributed under the terms of the Creative Commons Attribution 4.0 International License (http://creativecommons.org/licenses/by/4.0/), which permits use, duplication, adaptation, distribution, and reproduction in any medium or format, as long as you give appropriate credit to the original author(s) and the source, provide a link to the Creative Commons license, and indicate if changes were made.

The original article has been corrected.

Open Access This article is distributed under the terms of the Creative Commons Attribution 4.0 International License (http://creativecommons. org/licenses/by/4.0/), which permits unrestricted use, distribution, and reproduction in any medium, provided you give appropriate credit to the original author(s) and the source, provide a link to the Creative Commons license, and indicate if changes were made. The images or other third party material in this article are included in the article’s Creative Commons license, unless indicated otherwise in a credit line to the material. If material is not included in the article’s Creative Commons license and your intended use is not permitted by statutory regulation or exceeds the permitted use, you will need to obtain permission directly from the copyright holder. To view a copy of this license, visit http:// creativecommons.org/licenses/by/4.0/.

